# Rufinamide (RUF) suppresses inflammation and maintains the integrity of the blood–brain barrier during kainic acid-induced brain damage

**DOI:** 10.1515/biol-2021-0090

**Published:** 2021-08-25

**Authors:** Huaxu Yu, Bin He, Xu Han, Ting Yan

**Affiliations:** General Surgery Department, Changsha Hospital of Hunan Normal University, No. 70, Lushan Road, Changsha 410000, Hunan, China; Department of Clinical Skills Training Center of ZhuJiang Hospital, ZhuJiang Hospital of Southern Medical University, Guangzhou 510282, China

**Keywords:** RUF, neuronal damage, neuroinflammation, blood–brain barrier

## Abstract

Rufinamide (RUF) is a structurally unique anti-epileptic drug, but its protective mechanism against brain injury remains unclear. In the present study, we validated how the RUF protected mice with kainic acid (KA)-induced neuronal damage. To achieve that, a mouse epilepsy model was established by KA intraperitoneal injection. After Nissl staining, although there was a significant reduction in Nissl bodies in mice treated with KA, 40, 80, and 120 mg/kg, RUF significantly reduced KA-induced neuronal damage, in a dose-dependent manner. Among them, 120 mg/kg RUF was most pronounced. Immunohistochemistry (IHC) and western blot analysis showed that RUF inhibited the IBA-1 overexpression caused by KA to block microglia cell overactivation. Further, RUF treatment partially reversed neuroinflammatory cytokine (IL-1β, TNFα, HMGB1, and NLRP3) overexpression in mRNA and protein levels in KA mice. Moreover, although KA stimulation inhibited the expression of tight junctions, RUF treatment significantly upregulated expression of tight junction proteins (occludin and claudin 5) in both mRNA and protein levels in the brain tissues of KA mice. RUF inhibited the overactivation of microglia, suppressed the neuroinflammatory response, and reduced the destruction of blood–brain barrier, thereby alleviating the excitatory nerve damage of the KA-mice.

## 1 Introduction

Epilepsy, one of the most common severe brain diseases afflicting 70 million people worldwide, has a bimodal incidence, with infants and the elderly at the highest risk [1]. The common cause of epilepsy are excessive depolarization and increased excitability of neurons in the brain [2]. Glutamate is the primary excitatory neurotransmitter in the mammalian brain. Its overproduction can lead to excitatory toxicity and central neuron death, which are closely related to the onset of epilepsy [3]. Kainic acid (KA) is a glutamic acid analogue of excitatory toxicity, which can be used to simulate excitatory toxicity caused by over-activated glutamate [4]. Thus, KA is often used to induce recurrent animal models of spontaneous epilepsy. In addition, since the subzone of the hippocampal area is vulnerable to damage induced by KA, the KA-induced disease model is widely applied in the related scientific research of neurodegenerative diseases, such as epilepsy [5,6].

The occurrence of excitatory neurotoxicity is often accompanied by abnormal activation of microglia cells, which induces the release of a large number of pro-inflammatory factors. The neuroinflammatory response triggered by excitatory toxicity results in damage to hippocampal neurons [7]. Further, increased pro-inflammatory cytokines may aggravate neuron injury. The long-term activation of microglial cells caused by excitatory toxicity and subsequent secretion of inflammatory factors would amplify the inflammatory response in the central nervous system, increasing the risk of epilepsy [8–10]. In addition, long-term activation of microglia cells contributes to damage of the blood–brain barrier, allowing serum proteins to insinuate into the brain, which also increases the release of inflammatory cytokines. Correspondingly, the structure and foundation of the blood–brain barrier are disrupted, leading to more severe damage to the hippocampus [11,12]. By regulating the activation of microglia and maintaining the integrity of the blood–brain barrier, it may be a feasible method to reduce brain damage caused by epilepsy.

Rufinamide (RUF) is a uniquely structured anti-epileptic drug (AED) and blocks voltage-gated sodium channels (VGSCs). Based on its efficacy and behavioral toxicity profiles in animal seizure models, compared with other AEDs, RUF successfully treats generalized and partial seizures [13]. For example, in the occurrence of Lennox–Gastaut syndrome, RUF rapidly binds with inactivated sodium channels to inhibit spike and wave discharges, thereby inhibiting the seizure of such atypical epilepsy [14]. In addition, some previous studies have already reported the antioxidant and anti-inflammatory effects of RUF in various rodent seizure models [15]. Nevertheless, most of the research on RUF so far has focused on its effect on the frequency of seizures and the duration of seizure-like events [16]. The protective mechanism of RUF against brain injury induced by excitotoxicity remains unclear. This study intends to establish a mouse epilepsy model to evaluate the effects of RUF on microglia activation and BBB integrity in epileptic mice and to explore the mechanism of RUF on excitatory toxic brain injury.

## 2 Materials and methods

### 2.1 Establishment of the epilepsy model and experimental groups

All 30 male ICR mice (8 weeks old) were purchased from Guangdong Medical Laboratory Animal Center (Guangdong, China). The animals were housed in a conventional state under adequate temperature (23 ± 3°C) and relative humidity (55 ± 5%) control with a 12 h light/12 h dark cycle and provided free access to food and water. All animals were bred adaptively for one week before the experiment. A mice model of brain injury was established based on the previous study [3]. In short, KA (Sigma-Aldrich Company, CAS: 58002-62-3) was dissolved in sterile saline at 1 mg/mL, and 40 mg/kg KA was applied in the peritoneum of the model mice and the control mice were given sterile saline of the same volume. If the experimental animal dies after KA injection, the same number of experimental animals should be added.

Animals were divided into five groups (*n* = 6 in each group); [1] control group (sterile normal saline; 0.9% w/v NaCl), [2] KA-treated group (KA-group), [3] 40 mg/kg RUF-treated group (KA + low-RUF group), [4] 80 mg/kg RUF-treated group (KA + medium-RUF group), and [5] 120 mg/kg RUF-treated group (KA + high-RUF group). During the experiment, one mouse died after KA injection. RUF was administered intraperitoneally once daily for 3 days before KA injection, and the last administration of vehicle or RUF was performed 1 h before KA injection. The mice were euthanized 24 h after KA injection.

**Ethical approval:** The research related to animal use has been complied with all the relevant national regulations and institutional policies for the care and use of animals and was approved by the Ethics Committee of the Fourth Hospital of Changsha (approval number: CSSDSYY-LLSC-KYXM-2018-01-06).

### 2.2 Tissue sections

After mice were anesthetized, the thoracic cavity was opened to expose the heart, and then cardiac perfusion was performed with 100 mL of normal saline. When the lungs on both sides and the liver of the mice were obviously whitened, the brain tissues of the mice were taken and continuously fixed in 4% paraformaldehyde for 24 h, followed by gradient dehydration with 20 and 30% sucrose solutions, respectively. The brain tissue of the mice was sectioned, dehydrated, and embedded in paraffin. Finally, the tissue sections were placed on the slides and dried at 60°C for 2 h [17].

### 2.3 Nissl stain

Nissl staining was performed as previously described [17]. The sections were dewaxed and hydrated, and the sections were first placed in xylene 2 times (10 min/time). Then, 100, 95, 85, and 75% ethanol were placed at each stage for 5 min. Subsequently, the sections were soaked in distilled water for 5 min and then put into 0.1% toluidine blue solution (Amresco, Solon, OH, USA) staining solution for 5 min. After washing with distilled water, the sections were placed in ethanol at different concentration gradients (70% for 2 min; 90% for 5 min) until the nucleus and cell granules were transparent and the background became colorless. Then, the slides were immersed in xylene twice (10 min/time), sealed with neutral gum, and observed under a microscope (Motic, China). Brain slices from the control group of mice (CA1 region) were used as a positive control. The number of Nissl bodies was calculated by IPP (image-pro-Plus, Media Cybernetics, USA) software.

### 2.4 Immunohistochemistry

IHC assay was performed as described previously [4]. First, brain slices (CA1 region) were placed in xylene 3 times (20 min/time). Then, the slices were sequentially placed in 100, 95, 85, and 75% ethanol at each stage for 5 min. Afterward, 1% periodate was added to inactivate endogenous enzymes at room temperature for 10 min, and rinsed with PBS 3 times (3 min/time). Then, the primary antibody IBA-1 (1:200, Proteintech, USA) was incubated overnight at 4°C, and the secondary antibody IgG antibody-HRP polymer was incubated for 30 min at 37°C. Diaminobenzidine (DAB, ZSBG-BIO, China) was used to visualize the slices. After dehydration, the slices were sealed with neutral balsam and observed under a microscope (Motic, Guangzhou, China). The brain tissue slices from the control group of mice (CA1 region) were used as a positive control. Finally, IPP (image-pro-Plus, Media Cybernetics, USA) software was used for image analysis.

### 2.5 Reverse transcription quantitative polymerase chain reaction (RT-qPCR)

RT-qPCR was performed in accordance with a previous report [18]. Trizol reagent (Thermo, USA) was used to extract total RNA from fresh mice hippocampal tissues in each treatment group according to the instructions. The remaining tissue samples were stored at −80°C for subsequent research. The first-strand cDNA was synthesized from 500 ng of RNA using the HiFiScript cDNA Short Kit (Cowin Biosciences, China) for qPCR. All primers in this study were synthesized by Sangon Biotech Company (Shanghai, China). The reaction conditions were 95°C for 10 min, followed by 40 cycles at 95°C for 15 s and 60°C for 30 s. The normalization of the target genes used β-actin as the internal control. The 2^−ΔΔCt^ method was utilized to calculate the relative expression of the mRNA [19]. The primers for IL-1β, HMGB1, NLRP3, occludin, TNF-α, claudin5, and β-actin are listed in Table 1.

**Table 1:** The primer sequence of gene

Gene	Primer sequence (5′ → 3′)
IL-1β	FOR: TGTGATGTTCCCATTAGAC
	REV: AATACCACTTGTTGGCTTA
HMGB1	FOR: GGCGGCTGTTTTGTTGACAT
	REV: ACCCAAAATGGGCAAAAGCA
NLRP3	FOR: CACCTCTTCTCTGCCTACCTG
	REV: AGCTGTAAAATCTCTCGCAGT
Occludin	FOR: CCCAGACCACTATGAAACCGACT
	REV: CAGCCATGTACTCTTCGCTCT
TNF-α	FOR: CCCCTCTATTTATAATTGCACCT
	REV: CTGGTAGTTTAGCTCCGTTT
Claudin5	FOR: TGGCACTCTTTGTTACCTTGACC
	REV: ACCGTTGGATCATAGAACTCCC
β-Actin	FOR: ACATCCGTAAAGACCTCTATGCC
	REV: TACTCCTGCTTGCTGATCCAC

### 2.6 Western blot

The fresh hippocampal tissues of mice from different treatment groups were cleaved with RIPA lysis buffer and centrifuged at 12,000 rpm at 4°C for 15 min to obtain the sample proteins. The remaining tissue samples were stored at −80°C. Then, a BCA protein assay kit (Beyotime Biotechnology, China) was used to determine the concentration of proteins. Aliquots of 20 μg of protein of each sample were isolated by 12% sodium dodecyl sulfate-polyacrylamide gel electrophoresis (SDS-PAGE). The separated proteins were transferred to a polyvinylidene fluoride membrane activated by methanol, sealed by 5% skim milk, and dried at room temperature for at least 1 h. Afterward, it was incubated with the first antibody overnight at 4°C. The main incubated primary antibodies included anti-occludin (1:3,000, Proteintech, USA), anti-Claudin5 (1:750, Bioss Biotechnology Co., Ltd., Beijing, China), anti-IL-1B (1:500, Proteintech, USA), anti-TNFα (1:750, Proteintech, USA), anti-HMGB1 (1:750, Proteintech, USA), anti-NLRP3 (1:500, Proteintech, USA), and internal reference anti-β-actin (1:500, Proteintech, USA). The proteins were combined with secondary anti-IgG (1:5,000, 1:6,000, Proteintech, USA) and incubated at 37°C for 90 min, and later visualization was performed by the Millipore (USA) method, and imaging analysis was performed with ImageQuant TL software (GE Healthcare, Life Sciences, USA)[ 20].

### 2.7 CCK8

According to previous literature records, 0, 5, 10, and 20 μg/mL RUF were used to pretreat glial cells at 37°C and 5% CO_2_ for 24 h [21,22]. Cell Counting Kit-8 (Dojindo, Tokyo, Japan) was used to detect cell viability according to the manufacturer’s instructions.

### 2.8 Statistics

Graphpad Prism8.0 (GraphPad Software Inc., USA) was used for the statistical analysis of data in this study. Measurement data were expressed as mean ± standard deviation. Tests of normality and homogeneity of variance were first performed to verify that they were in accordance with normal distribution and homogeneity of variance. Non-paired *t*-test was used for inter-group comparison, one-way ANOVA was used for inter-group comparison, and Tukey’s was used for post-test. *P* < 0.05 indicated that the difference was statistically significant.

## 3 Results

### 3.1 RUF reduced hippocampal neuronal injury in the KA mice model

**Figure 1:**
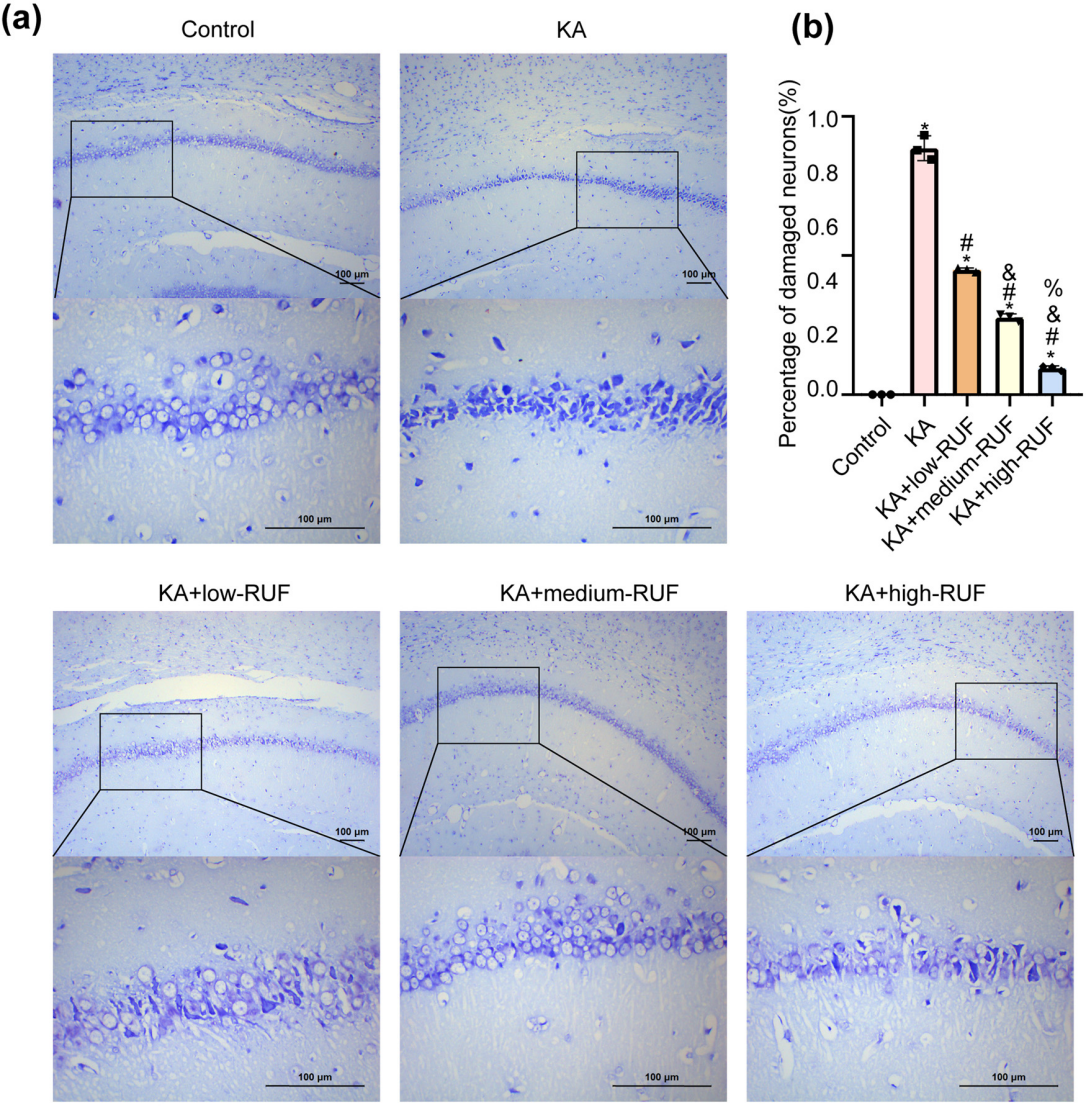
RUF reduced hippocampal neuronal injury in the KA mice model. (a) Nissl staining was used to analyze the distribution of hippocampal neurons damage in mice. (b) The percentage of damaged hippocampal neurons was analyzed with Nissl staining. ^*^Compared with control group, *P* < 0.05. ^#^Compared with the KA group, *P* < 0.05. ^&^Compared with KA + low-RUF group, *P* < 0.05. ^%^Compared with KA + medium RUF group, *P* < 0.05.

When neurons are damaged, the Nissl bodies would decrease, disintegrate, or even disappear [23]. Nissl staining analysis (Figure 1a) showed that the hippocampal neurons were tightly arranged in the control group, the cell structure was clear, and Nissl bodies were abundant in the cytoplasm. In the KA group, the hippocampal neurons were loosely arranged, with blurred cell contour and atrophy, and the number of Nissl bodies in the cytoplasm was significantly reduced. These results indicated that hippocampal neurons in the KA group were severely damaged. Further, in RUF treatment, we found that the status of hippocampal neurons was reversed markedly in a dose-dependent manner. The statistical results of the percentage of damaged neurons were consistent with the results of Nissl staining. As shown in Figure 1b, with the induction of KA, the proportion of injured neurons increased. In the RUF treatment group, the proportion of damaged cells was significantly reduced in a dose-dependent manner. The data illustrated that RUF could alleviate KA-induced neuronal injury. Since the best effect was displayed by the high-dose RUF (120 mg/kg RUF) group, it was selected for the subsequent experimental study.

### 3.2 RUF suppressed microglial activation in the KA mice model

**Figure 2:**
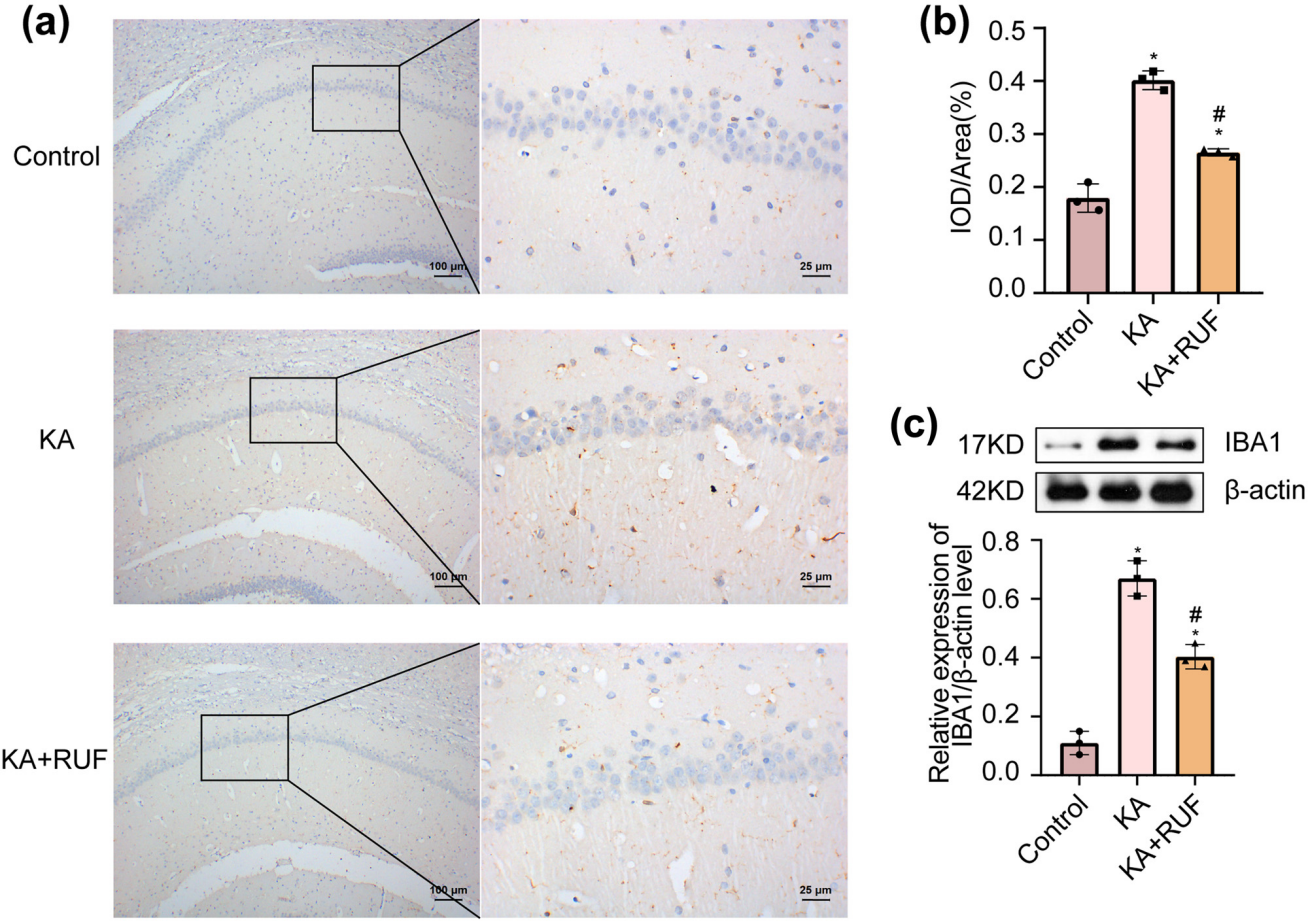
Expression of microglia marker IBA-1 protein in mice. (a) IHC analysis of the expression of IBA-1 protein in mouse brain. (b) IHC analysis of the IOD/area of IBA-1 protein in mouse brain. (c) Western blot analysis of the relative expression of IBA-1 protein in mouse brain. (d) Cell viability was analyzed by CCK8. ^*^Compared with control group, *P* < 0.05. ^#^Compared with the KA group, *P* < 0.05. IOD: integrated optical density (brown = immunostaining, blue = nucleus).

Excitatory neurotoxicity is implicated in the abnormal activation of microglia. Thereby, we further analyzed the expression of IBA-1, a microglial marker in the central nervous system, by immunohistochemistry. In Figure 2a and b, the results showed that compared with the control group, the IBA-1 IOD/area in the brain of the KA group was significantly increased. Compared with the KA group, the IBA-1 IOD/area in the KA model mice was significantly reduced after RUF intervention. It was also confirmed by western blot analysis. From Figure 2c, the relative expression level of IBA-1 protein in the brain tissue of the KA group was significantly higher than that of the control group. Compared with the KA group, the relative expression of IBA-1 protein in the KA model mice was significantly reduced after RUF intervention. In order to investigate whether RUF had an effect on microglial cell viability, we treated microglia cells with 5, 10, and 20 μg/mL RUF for 24 h. There was no significant difference in microglia viability between the RUF treatment group and the control group (*P* < 0.05) (Figure 2d). The above results indicated that RUF could inhibit the excessive activation of microglia cells in the KA-induced mice rather than inhibiting cell proliferation.

### 3.3 RUF inhibited the expression of inflammatory factors in the KA mice model

**Figure 3:**
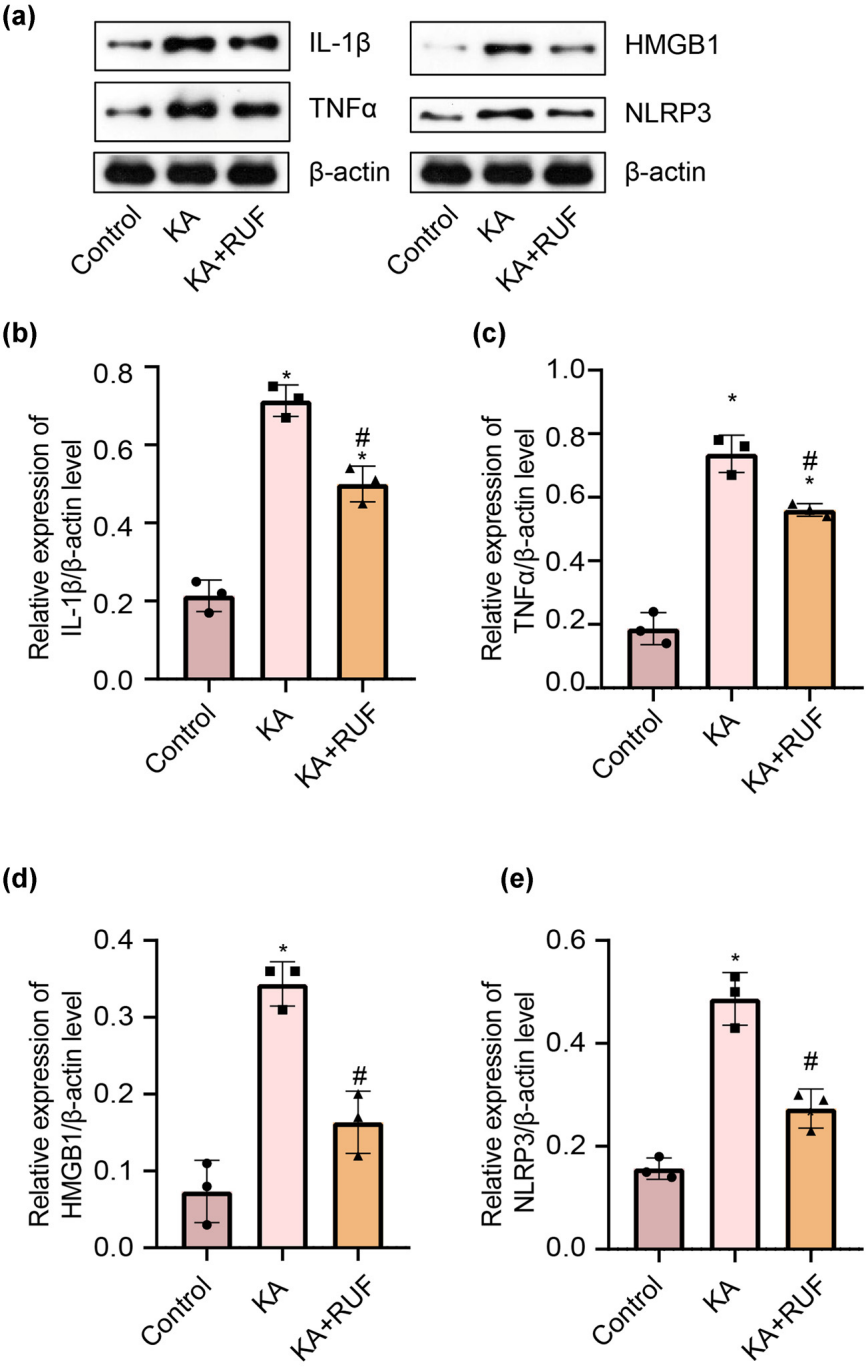
Western blot analysis of IL-1β, TNFα, HMGB1, and NLRP3 in mice. (a) Western blot bands about IL-1β, TNFα, HMGB1, and NLRP3. (b) The relative expression of IL-1β/β-actin at the protein level. (c) The relative expression of TNFα/β-actin at the protein level. (d) The relative expression of HMGB1/β-actin at the protein level. (e) The relative expression of NLRP3/β-actin at the protein level. ^*^Compared with control group, *P* < 0.05. ^#^Compared with the KA group, *P* < 0.05.

**Figure 4:**
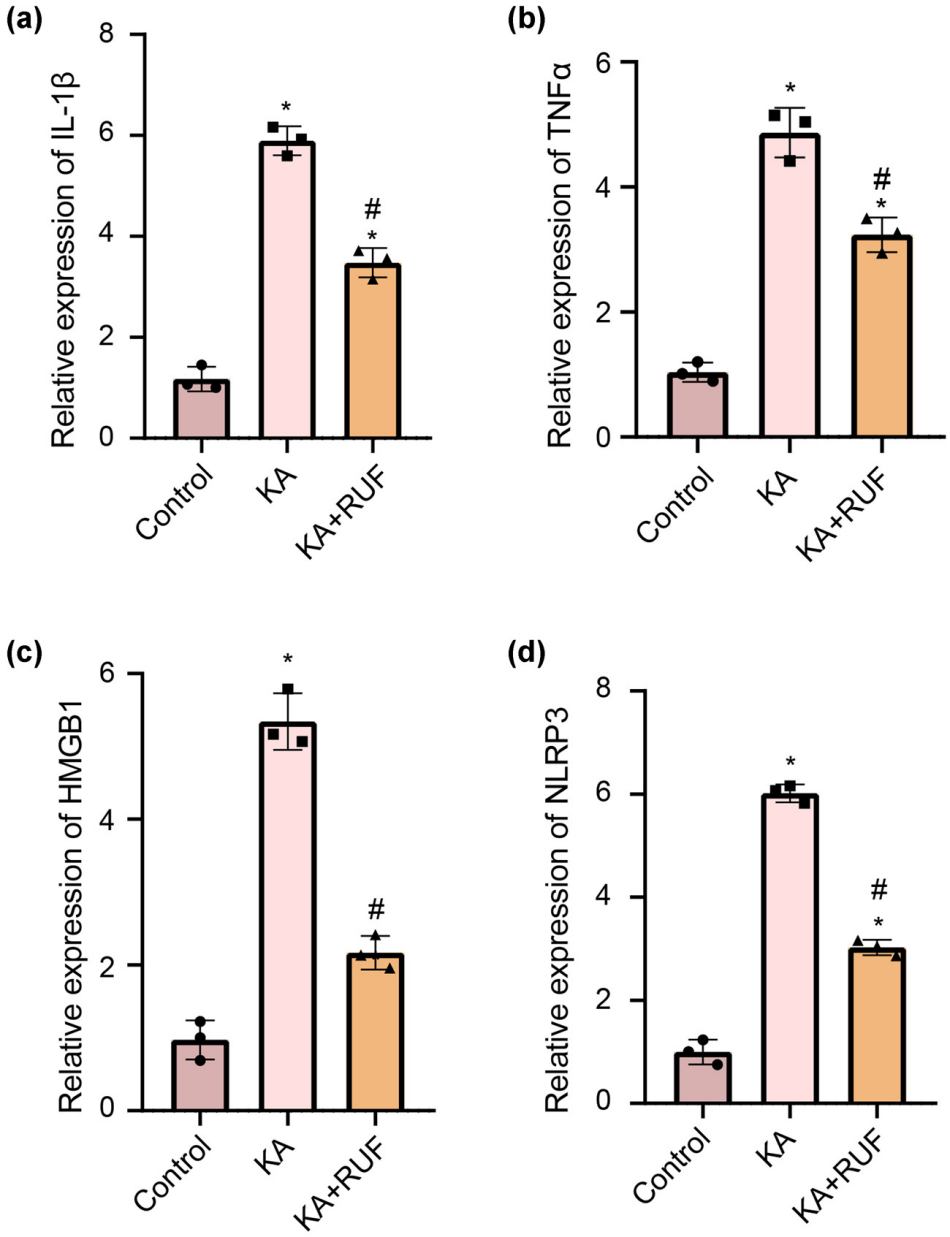
RT-qPCR analysis of IL-1β, TNFα, HMGB1, and NLRP3 in mice. (a) The relative expression of IL-1β mRNA in different groups. (b) The relative expression of TNFα mRNA in different groups. (c) The relative expression of HMGB1 mRNA in different groups. (d) The relative expression of NLRP3 mRNA in different groups. ^*^Compared with control group, *P* < 0.05. ^#^Compared with the KA group, *P* < 0.05.

In order to study the effect of RUF on neuroinflammation caused by KA-induced brain injury, we performed a western blot to detect the expression of neuroinflammation-related factors in the brain tissue of mice at the protein level. The results in Figure 3a–d showed that compared with the control group, the expression of IL-1β, TNFα, HMGB1, and NLRP3 in the brain tissues of mice in the KA group were significantly increased. However, this state in neuroinflammatory cytokines was partially reversed due to the treatment of RUF. Consistent with protein levels, these inflammatory cytokines, which were highly expressed at the mRNA transcription level due to KA stimulation, were down-regulated with RUF intervention (Figure 4a–d). Herein, the findings revealed that RUF has a certain positive effect on inhibiting KA-induced neuroinflammation in mice.

### 3.4 RUF attenuated the blood–brain barrier injury in the KA mice model

**Figure 5:**
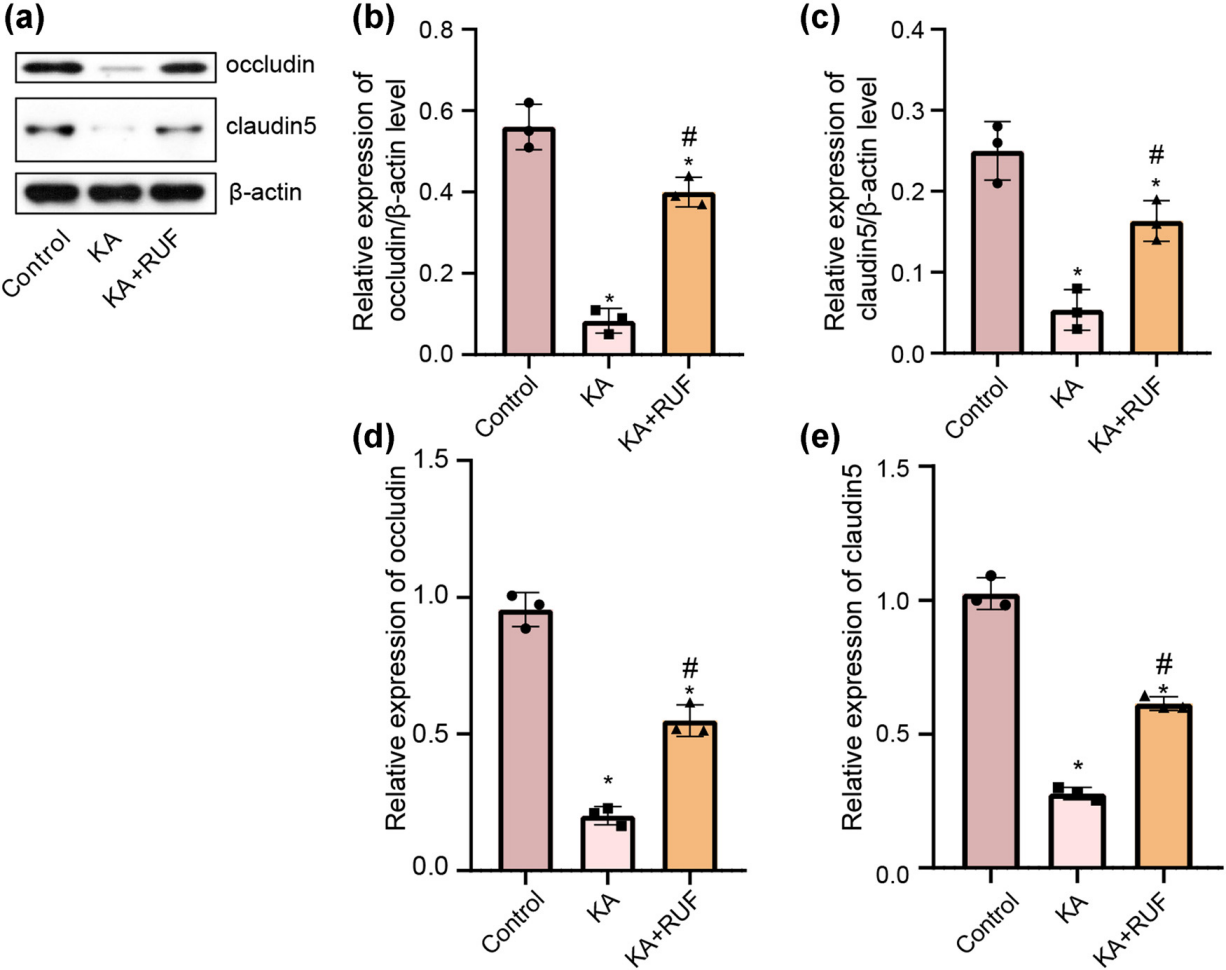
Western blot and RT-qPCR analysis occludin and claudin 5 protein in mice. (a) Western blot bands about occludin and claudin 5 in mice brain. (b) The relative expression of the occludin/β-actin protein in mice brain. (c) The relative expression of claudin 5/β-actin protein in mice brain. (d) The relative expression of occludin mRNA in mice brain. (e) The relative expression of claudin 5 mRNA in mice brain. ^*^Compared with control group, *P* < 0.05. ^#^Compared with the KA group, *P* < 0.05.

The rupture of the blood–brain barrier is a sign of central nervous system dysfunction. To investigate whether RUF could protect the blood–brain barrier from KA-induced damage, we performed western blot to measure the protein expression of tight junction proteins occludin and claudin 5 in the brain tissue of mice. As shown in Figure 5a, compared with the control group, the 5 bands of occludin and claudin 5 in the KA group developed weaker. In contrast, the development of both bands increased significantly in the RUF group. The quantitative analysis of western blot results showed that RUF significantly increased the expression of occludin and claudin 5 at the protein level (Figure 5b and c). The results of RT-PCR also agree with this result. At the mRNA level, RUF treatment reversed the low expression of occludin and claudin 5 induced by KA stimulation (Figure 5d and e). From above, it is suggested that RUF has the potential to protect KA-mice against blood–brain barrier injury.

## 4 Discussion

Neurotoxicity, neuronal cell death, neuroinflammation, oxidative stress, gliosis, and BBB dysfunction are all common causes of epilepsy [24]. In recent years, the treatment of epilepsy is still based on drug therapy, such as RUF [25]. RUF is a triazole derivative whose main mechanism of action is considered the regulation of sodium channel activity, especially the prolongation of sodium channel inactivation, which can be used in the adjuvant treatment for patients with epilepsy [26]. Many studies have shown that RUF is well tolerated and safe. For example, RUF effectively suppressed tonic-clonic seizures caused by maximum electroshock in rodents, either orally or intraperitoneally [27]. As a marker of the functional state of neurons, Nissl bodies are often used to evaluate the changes of neurons [23]. In this study, we observed a dose-dependent reversal of the KA-stimulated reduction in the number of Nissl bodies after 40, 80, and 120 mg/kg RUF treatment. It implied RUF could effectively prevent KA-induced damage to mice hippocampus neurons. Herein, we further hypothesized that this condition might be associated with microglia activation.

Emerging evidence has suggested that KA can cause characteristic neuronal hippocampal subregion damage in young rats, accompanied by accumulation of microglia [28]. In our research, RUF showed an excellent inhibitory effect on the KA-induced overactivation of microglial cells, rather than inhibiting the cell viability of microglia. Over the past decade, various experimental studies have shown that glial cells activated by various lesions play an essential role in the mechanism of epileptic seizure and relapse [29]. Pro-inflammatory molecules alter neuronal excitability and glial cell physiological function through paracrine or autocrine function, which would contribute to a disturbance in glial neuron communication or even recurrent ischemic attacks [30]. Other reports have shown that microglia act as innate immune effector cells in the central nervous system and secrete inflammatory factors to amplify the inflammatory response [31]. Microglia may be one of the sources of this secretion, which needs further research and analysis. As this is not the focus of the current study, we will explore this topic in future work.

After a seizure, inflammatory mediators released by brain cells and peripheral immune cells may cause various seizures and persistent seizures [32,33], such as IL-1β, TNFα, HMGB1, IL6, IL8, NLRP3 and other related factors [34,35]. Therefore, we speculate that RUF may also play a positive role in controlling inflammation in epileptic animals. We found that inflammatory cytokines expressions were all increased significantly with the overactivation of microglial cells in KA mice. In this study, the inhibitory rates of RUF on the inflammatory proteins IL-1β, TNF-α, HMGB1, and NLRP3 were 29.9, 24.0, 52.4, and 43.8%, respectively. The inflammatory response of KA + RUF group mice was effectively alleviated. It is suggested the RUF has a certain positive effect on inhibiting KA-induced neuroinflammation in mice. This view is also supported by another study [4]. Microglia, a neuroinflammatory effector in the brain, belongs to the central nervous system and is regulated by the vagus nerve to modulate its inflammatory state [36]. Further, we hypothesized that RUF might exert anti-inflammatory effects through some central nervous or autonomic nervous functions. In the next step, we will compare the anti-inflammatory effects of RUF with classic anti-inflammatory compounds and explore its specific mechanisms.

The integrity of BBB maintains the optimal microenvironment for brain function [37]. BBB dysfunction has been identified as a critical factor in several neurological diseases, including epilepsy and stroke. In Alzheimer’s disease, Avarol derivatives (such as thiosalycil-prenyl-hydroquinones) act as competitive acetylcholinesterase (AChE) inhibitors and show good neuroprotection [38,39]. Such inhibitors reduce the consumption of acetylcholine by AChE, which may reduce BBB promiscuity [40]. As far as we know, RUF does not interact with acetylcholine. In our study, although tight junction proteins (occludin and claudin 5) expression were blocked in the brain tissue of KA-mice, RUF restored both partly. It manifested that RUF has a protective effect on the integrity of BBB. The preservation of BBB integrity may be the main reason for its neuroprotective effect. Tight junction proteins bind endothelial cells to each other, resulting in a barrier that prevents most molecules from penetrating through the cross-cellular pathway [41]. Perampanel, an AED similar to RUF, acts as a non-competitive AMPA receptor antagonist and provides a protective effect against ischemic stroke through claudin 5-mediated permeability regulation of BBB [42]. On the other hand, the considerable accumulation of inflammatory factors is also a major cause of BBB destruction. Novel research found that Escin reduced BBB damage by inhibiting systemic inflammatory response in mice with cerebral hemorrhage, which led to improved neurological function [43]. Therefore, we supposed that the inhibitory effect of RUF on neuroinflammation in KA mice might protect BBB from damage to a certain extent. In addition, it is worth noting that overexpressed P-glycoprotein (PGP) exports AED in BBB may be one of the causes of AED resistance. Although RUF is not the substrate of PGP that cannot penetrate the BBB [44], RUF may activate the transcriptional levels of both through specific mechanisms or pathways, thus increasing the mRNA expressions of occludin and Claudin5. Furthermore, mRNAs were translated into the corresponding proteins, so as our results showed, occludin and claudin5 also appeared corresponding changes in protein levels. This pharmacological feature may reduce its role in the destruction of BBB and may be related to its long-term efficacy. However, this characteristic may also be a disadvantage of RUF treatment.

However, we must admit that this study has some limitations. Due to our current experimental conditions and funding constraints, the sample size used in this study is small, which leads to an insufficient exploration of some findings. In terms of controlling inflammation, we found that RUF has a positive effect on inhibiting inflammation. This interesting phenomenon aroused our interest. We plan to explore the possible mechanism of this phenomenon in the next research. At the same time, based on the protective effect of RUF on the integrity of BBB, we plan to conduct in-depth research on the mechanism of this process from the perspective of cell or blood metabolism.

## 5 Conclusion

In conclusion, our current study demonstrates that RUF could inhibit the overactivation of microglial cells. It also has a positive effect on preventing the overexpression of inflammatory cytokines. At the same time, RUF reduces the damage of BBB integrity caused by KA. These may be essential pathways by which RUF protects KA mice from excitatory nerve damage. These may be connected with the neuroprotective effect of RUF. Our study may provide insights better to understand the regulatory mechanism of RUF on brain injury.
